# A novel spontaneous hepatocellular carcinoma mouse model for studying T-cell exhaustion in the tumor microenvironment

**DOI:** 10.1186/s40425-018-0462-3

**Published:** 2018-12-07

**Authors:** Yu-Tzu Liu, Tai-Chung Tseng, Ruey-Shyang Soong, Chun-Yi Peng, Yu-Hsing Cheng, Shiu-Feng Huang, Tsung-Hsien Chuang, Jia-Horng Kao, Li-Rung Huang

**Affiliations:** 1 Institute of Molecular and Genomic Medicine, National Health Research Institutes, No.35, Keyan Road, Zhunan Town, Miaoli County 350 Taiwan; 2 Division of Gastroenterology and Hepatology, Department of Internal Medicine, National Taiwan University Hospital, Taipei, Taiwan; 3 Hepatitis Research Center, National Taiwan University, National Taiwan University Hospital, Taipei, Taiwan; 4 Department of General Surgery, Chang Gung Memorial Hospital, Keelung, Taiwan; 5 College of Medicine, Chang Gung University, Taoyuan, Taiwan; 6 Immunology Research Center, National Health Research Institutes, Miaoli, Taiwan; 7 Graduate Institute of Clinical Medicine, National Taiwan University, Taipei, Taiwan; 8 Department of Medical Research, National Taiwan University, National Taiwan University Hospital, Taipei, Taiwan

**Keywords:** Hepatocellular carcinoma, T-cell exhaustion, Tumor-associated macrophage, MDSC, Tumor-specific T cell, Immune checkpoint, Immunotherapy

## Abstract

Immunotherapy has ushered in a new era of cancer therapy, and this is also applicable to therapy of hepatocellular carcinoma (HCC). In this context, effective development of therapeutic strategies requires an HCC mouse model with known tumor-associated antigens (TAAs) and an HCC growth reporter. We created such a model using hydrodynamic injection and a transposon system to introduce *AKT* and *NRAS* and open reading frames (ORFs) encoding surrogate tumor antigens and luciferase into chromosomes of hepatocytes to induce nodular and diffuse tumors in the liver. TAA-specific CD8^+^ T cells were detected during HCC progression; however, these showed exhausted-like phenotypes and were unable to control tumor growth. Myeloid-derived suppressor cells (MDSCs) and tumor-associated macrophages (TAM) from the tumor microenvironment were found to contribute to the suppression of the CD8^+^ T-cell response. The transposon-based Akt/N-Ras-induced HCC mouse model we developed enables researchers to monitor tumor growth non-invasively and to quantify and characterize endogenous or adoptively transferred TAA-specific CD8^+^ T-cell responses. These features make it a suitable preclinical model for exploration and evaluation of immune checkpoint inhibitors and cell-based immunotherapies for HCC treatment.

**Electronic supplementary material:**

The online version of this article (10.1186/s40425-018-0462-3) contains supplementary material, which is available to authorized users.

## Background

Hepatocellular carcinoma (HCC) is the second leading cause of cancer deaths worldwide. HCC responds poorly to chemotherapies or targeted therapies and therefore developing new therapeutic strategies for HCC treatment, especially immunotherapies, is now on the rise [1]. The breadth of tumor-associated antigen (TAA)-specific CD8^+^ T-cell responses has been found to be positively correlated with HCC patient survival [2]. Therefore, immunotherapies for treatment of malignancies e.g. immune checkpoint inhibitors or adoptive T-cell therapy aim to restore TAA-specific T-cell responses. A suitable immunocompetent HCC mouse model for studies on HCC tumor immunology and immunotherapy development is urgently needed. There are various experimentally induced liver tumor mouse models including chemically induced models, implantation models (syngeneic and xenograft model) and genetically engineered models (GEM) [3, 4]. The immunocompromised xenograft HCC models are not ideal for use in immunological studies, whereas humanized mice with a functional human immune system may address this drawback. Although syngeneic HCC implants can grow in immunocompetent mice, the initial immune response in the tumor microenvironment (TME) may not mimic that arising from spontaneous HCC. Most of the chemically induced models or GEM require a long period of time to develop tumors and it can be challenging to monitor tumor growth in such mice.

Hydrodynamic injection (HDI), designed to deliver naked DNA into hepatocytes, is widely used in studies of liver diseases including persistent HBV infection and HCC [5, 6]. Combining HDI and a transposon system to deliver oncogenes could facilitate transfection and transformation of hepatocytes, making the technique perfect for establishment of various kinds of HCC models and to examine the oncogenic potential of specific genes in liver cancers. The advantage of HDI-based HCC mouse models over GEMs is their flexibility in terms of transgenes and strains of the recipient mice, reducing cost and time. Neuroblastoma Ras viral oncogene homologs (*Nras, NRasV12*), *shP53*, *myr-AKT*, c-*Myc*, *ΔN90-β-catenin*, c-*Met* and other oncogenes or viral genes have been used to establish HDI-based HCC models [6]. The time requirement for HCC growth in these HDI-based models is much less than other viral gene-transgenic (tg) mouse models e.g. HBx, HBs models.

Delivery of activated forms of *AKT* and *Nras* via a transposon system into mouse hepatocytes has been shown to induce rapid HCC growth in FVB/N mice [7]. Although activating Ras mutations are seldom found in human HCC samples, simultaneous activation of Akt/mTOR and Ras/MAPK pathways is often found in human HCC [8]. Previous studies examining the potential and roles of *AKT* and *RAS* in HCC induction have shown that activated *AKT* alone required nearly 30 weeks to induce HCC formation [9] whereas activated *RAS* alone was not able to induce HCC formation but caused hepatocyte senescence in immunocompetent mice [10]. The Akt/mTOR pathway involves in lipogenesis, which also promotes the development of HCC [9, 11]. We therefore adopted the Akt/N-Ras-based HDI technology [7] to establish a novel HCC mouse model expressing luciferase and surrogate tumor antigens (Ags) to monitor tumor growth non-invasively. Tumor progression in this HCC model was found to be more rapid than that in most of the chemically induced and genetically modified models. Both diffuse and nodular types of HCC were observed to develop in this model. We were able to characterize the exhausted state of TAA-specific CD8^+^ T cells and immunosuppressive cell populations in the TME in the model, indicating that it can be a suitable preclinical model for exploration and evaluation of immune checkpoint inhibitors and cell-based immunotherapies for HCC treatment.

## Methods

### Animal studies and hydrodynamic injection

Male C57BL/6j mice at the age of 4–5 week-old were purchased from the National Laboratory Animal Center (Taipei, Taiwan) and were kept in laboratory animal center (LAC) of NHRI. HBc_93–100_-specific T cell receptor (TCR) tg mice [12] were kindly provided by Dr. Francis V. Chisari and Dr. Masanori Isogawa (The Scripps Institute, La Jolla, USA) and were kept in LAC of NHRI. The two animal facilities are accredited by Association for Assessment and Accreditation of Laboratory Animal Care International (AAALAC International). C57BL/6j mice were anesthetized by Isoflurane mixed with O_2_ before HDI and given HDI of endotoxin-free plasmids dissolved in filtered Dulbecco Phosphate Buffered Saline (DPBS) in a volume equivalent to 8% body weight within 5 s. For the mice receiving 2 μg of pCMV(CAT)T7-SB100, 10 μg of pT/Caggs-NRASV12 and 10 μg of pKT2/CLP-AKT-LUC or pKT2/CLP-AKT-2A-OVA-HBc-HBs-LUC plasmids, the photons emitted from the transduced hepatocytes or tumor cells within the live animals were detected and quantified periodically using IVIS imaging system (Caliper Life Sciences, Massachusetts, USA). The mice were injected intraperitoneally with 3 mg of D-luciferin (Biosynth Chemistry & Biology, Staad, Switzerland) and waited for 10 min before being imaged under anesthesia by isoflurane inhalation. HCC-bearing mice with the total flux from IVIS imaging above 3 × 10^10^ photons/sec were subjected to human sacrifice to avoid the suffering of mice from large liver tumors.

### Construction and plasmid preparation

**Fig. 1:**
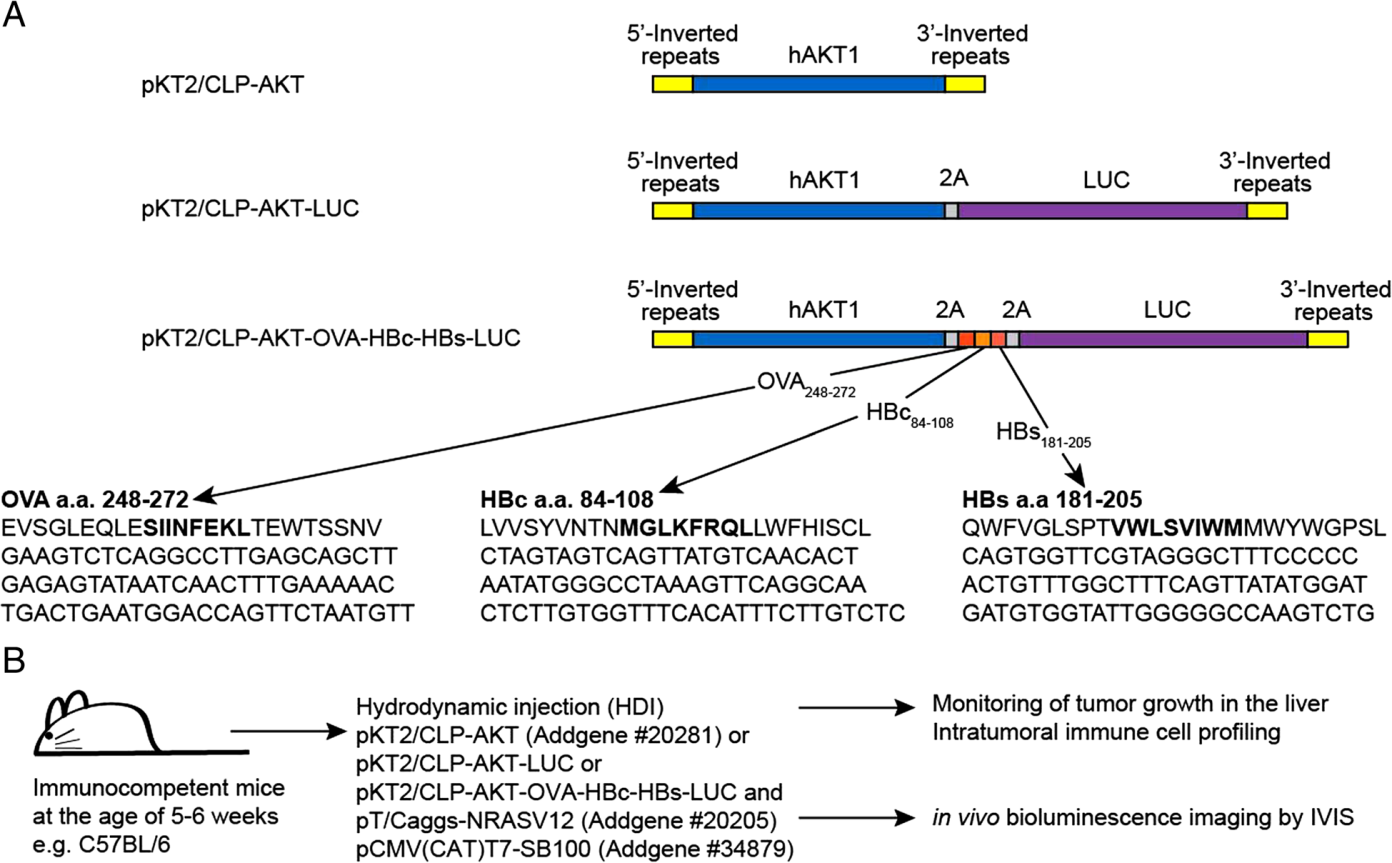
**a** Schematic representation of transposon constructs used for induction of HCC contain 5′ and 3′ inverted repeats, human AKT1 (hAKT) gene, p2A linker peptide sequence (2A), firefly luciferase reporter (LUC) gene and a mini-gene encoding ploypeptides derived from ovalbumin, HBV core protein and HBV surface protein (OVA-HBc-HBs). **b** Experimental scheme of HCC induction and monitoring

pCMV(CAT)T7-SB100 [13] was a gift from Zsuzsanna Izsvak (Addgene plasmid #34879). pKT2/CLP-AKT and pT/Caggs-NRASV12 [14] were gifts from John Ohlfest (Addgene plasmids #20281 & #20205). The ORF of firefly luciferase was amplified from pLAS5W-Luc-2A-eGFP [15] by PCR and sub-cloned into 3′ end of human *AKT1* of pKT2/CLP-AKT with p2A peptide sequence between the two ORFs to result in pKT2/CLP-AKT-LUC plasmid. The expression cassette was flanked by 5′ and 3′ transposon inverted repeats (IRs). A mini-gene encoding polypeptides derived from ovalbumin (OVA), core protein (HBcAg) and surface protein (HBsAg) of hepatitis B virus was synthesized (PURIGO Biotechnology, Taipei, Taiwan) and sub-cloned into pKT2/CLP-AKT-LUC to result in pKT2/CLP-AKT-OVA-HBc-HBs(Ags)-LUC plasmid. The DNA and polypeptide sequences of the mini-gene were listed in Fig. 1a. All the plasmids were amplified in DH5α *E.coli* and purified using QIAGEN EndoFree plasmid kits (Qiagen, Hilden, Germany).

### Immunohistochemistry study

Paraffin-embedded liver/tumor tissue sections were deparaffinized, rehydrated, followed by heat-induced antigen retrieval and then incubated with primary antibodies as listed in Additional file 1: Table S1. ImmPRESS anti-Rat Ig, ImmPRESS anti-Rabbit Ig, Mouse adsorbed (peroxidase) Polymer Detection Kit, DAB Peroxidase Substrate Kit and Hematoxylin (all from Vector laboratories, Burlingame, USA) were used for detection and visualization. For detection of CD45.1^+^ adoptively transferred CD8^+^ T cells, 5–7 μm of liver/tumor tissue frozen sections were fixed in ice cold acetone/chloroform (1:1), incubated with FITC-conjugated anti-CD45.1 (A20, Biolegend) and anti-FITC conjugate HRP (Thermo Fisher Scientific, Waltham, USA), followed by incubation with DAB substrate. For detection of lipid droplets, 5–7 μm liver/tumor tissue frozen sections were fixed in 10% formalin, incubated with oil red O dye for 15 min and washed with 60% isopropanol to remove the excess dye before mounting with glycerol jelly mounting medium. The images were captured using an automatic digital slide scanner Pannoramic MIDI with Plan-Apochromat 20x/0.8 objective (3D HISTECH) by Pathology Core Laboratory of NHRI and have been evaluated by an experienced pathologist (SFH).

### T-cell preparation and adoptive transfer

Splenic CD8^+^ T cells from CD45.1^+^ HBc_93–100_-specific TCR tg mice were isolated by immunomagnetic separation using CD8 microbeads (StemCell Technologies, Vancouver, Canada). Purified CD8^+^ T cells were stimulated for 3 days with anti-CD3/CD28 labeled T-activator Dynabeads® (Thermo Fisher Scientific) in RPMI 1640 medium (Thermo Fisher Scientific) supplemented with 8% FCS, 50 μM 2-mercaptoethanol, glutamine and antibiotics. After magnetic removal of Dynabeads, living cells were separated from dead cells using Ficoll-Paque plus density gradient media (GE healthcare Life Sciences, Illinois, USA). 2-3 × 10^5^ activated CD8^+^ T cells were adoptively transferred into HCC-bearing mice (CD45.2^+^) with total flux ranging from 1 × 10^8^ to 5 × 10^9^ photons/sec. The mice receiving adoptive transfer of HBc_93–100_-specific CTLs were given anti-mouse PD-1 Ab (clone: RMP1–14, Bio X cell, New Hampshire, USA) and isotype control Ab (clone: 2A3, Bio X cell), respectively, at the dose of 10 μg/gram body weight via intraperitoneal injection every 3 days for 6 times. The adoptively transferred CD8^+^ T cells could be visualized using a fluorescently labeled anti-CD45.1 antibody followed by flow cytometric analysis or immunohistochemical staining.

### Cell isolation and flow cytometry analysis

Livers were perfused via the portal vein with a 50 μg/ml collagenase IV solution (Sigma-Aldrich, St. Louis, USA), mechanically disrupted, and digested for 20 min at 37 °C in Gey’s balanced salt solution (GBSS) with 50 μg/ml collagenase IV, then filtered through a 250-μm cell strainer. Cells were re-suspended in 10 mL DPBS and underlaid with 5 mL of Ficoll-Paque plus density gradient media for gradient centrifugation for 10 min at 2000 rpm at 4 °C. After centrifugation, leukocytes were collected from the interface and subjected to flow cytometric analysis or FACSorting.

All stainings for flow cytometric analysis were performed in the presence of 10 μg/mL of Fc block (2.4G2) in fluorescence-activated cell sorting buffer (FACS buffer, phosphate-buffered saline/2% bovine serum albumin/0.02%NaN_3_). Acquisition and data analysis were conducted on Attune NxT flow cytometer (Thermo Fisher Scientific) and FlowJo software (V.10.0.8r1, FlowJo, LLC, Ashland, USA). Antibodies and dye used for flow cytometric analysis were listed in Additional file 1: Table S2. APC-conjugated dextramers recognizing H-2k^b^-restricted TCRs specific for OVA_257–264_ peptide (SIINFEKL), HBc_93–100_ peptide (MGLKFRQL) and HBs_190–197_ peptide (VWLSVIWM), respectively were purchased from Immudex (Copenhagen, Denmark). The cell number of specific cell populations in tumor and liver were adjusted to weight and expressed as × 10^6^ or × 10^3^/gram. The tumor-associated macrophages (TAMs) (CD146^−^CD11b^+^F4/80^+^MHC II^+^) and myeloid-derived suppressor cells (MDSCs) (CD146^−^CD11b^+^Gr-1^+^MHC II^−^) from liver tumors of HCC-bearing mice and macrophages (CD11b^+^F4/80^+^MHC II^+^) and Gr-1^+^ cells (CD11b^+^Gr-1^+^MHC II^−^) from spleens of normal C57BL/6j mice were isolated from tumor-associated leukocytes or splenocytes through FACSorting using Influx (BD Biosciences, San Jose, CA, USA).

### In vitro T-cell suppression assay

8 × 10^4^ anti-CD3/anti-CD28 beads activated HBc_93–100_-specific CD8^+^ T cells were re-stimulated by anti-CD3/anti-CD28 beads and were co-cultured with various numbers of FACSorted TAMs or MDSCs for 24 h. The proliferation of CD8^+^ T cells was measured by incorporation of 5-Ethynyl-2′-deoxyuridine (EdU), a thymidine analog. EdU (4 μM) was added at 6 h prior harvest of cells. EdU staining was performed using Click-iT EdU Alexa Fluor 488 Flow Cytometry Kit (Thermo Fisher Scientific).

### Statistic analysis

GraphPad Prism 7 (GraphPad Software, La Jolla, USA) and Student’s *t*-test were used for statistical analysis.

## Results

### Myristoylated (Myr)-Akt1 and N-Ras work synergistically to induce hepatocellular carcinoma in immunocompetent mice

We amplified and inserted a DNA fragment encoding the firefly luciferase reporter gene into the pKT2/CLP-AKT plasmid to result in the pKT2/CLP-AKT-LUC plasmid (Fig. 1a). Mini-gene encoded polypeptides derived from OVA (a.a. 248–272), and hepatitis B core (a.a. 84–108) and envelope (a.a. 181–205) proteins were synthesized and inserted into the pKT2/CLP-AKT-LUC plasmid to result in the pKT2/CLP-AKT-OVA-HBc-HBs- LUC plasmid (Fig. 1a). The amino acids in bold in each polypeptide are epitopes presented by MHC class I-K^b^. The epitopes SIINFEKL and MGLKFRQL could be recognized by TCRs of OT-I tg mice and HBc_93–100_ TCR tg mice, respectively (Fig. 1a).

**Fig. 2:**
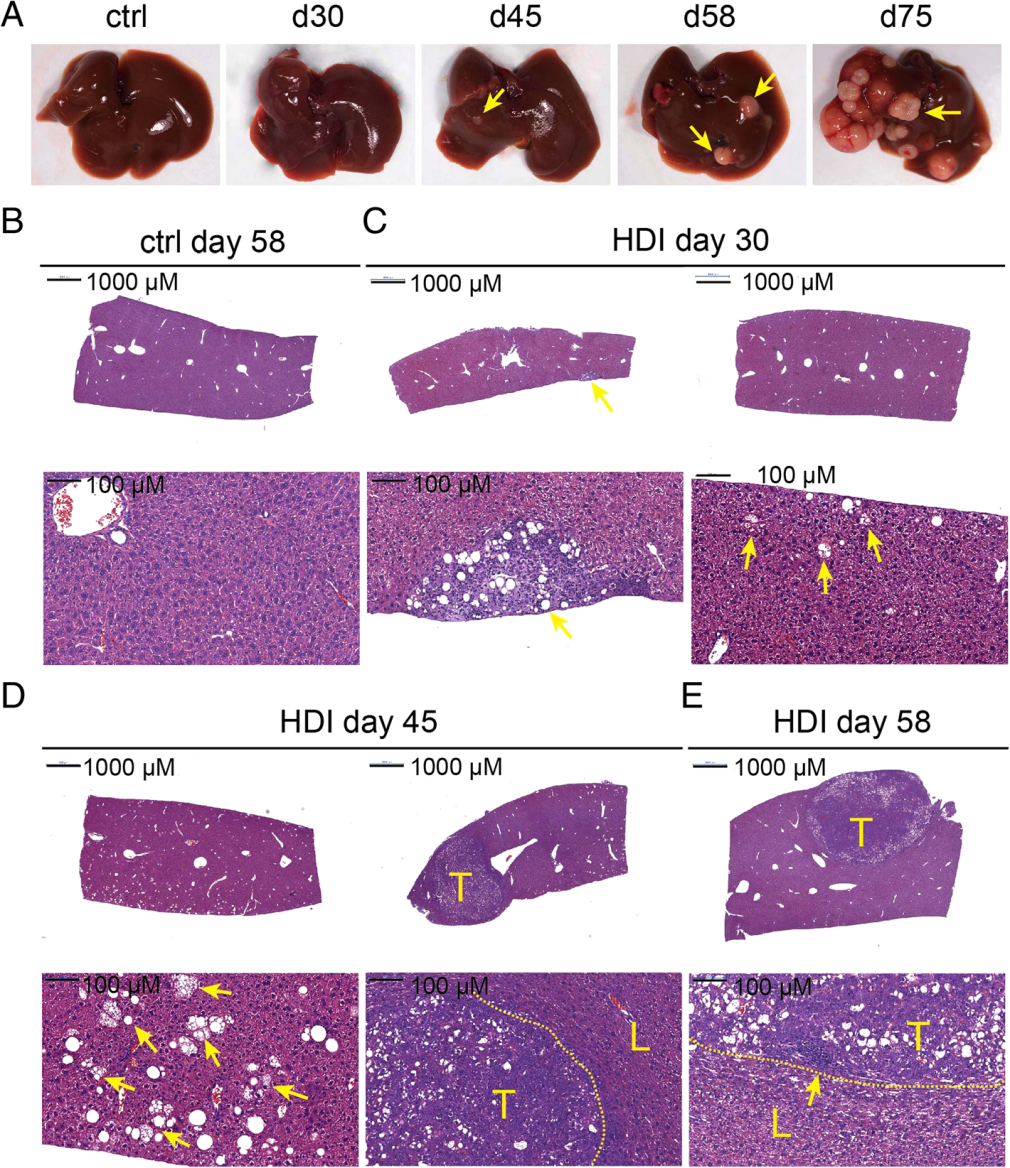
Kinetics of HCC induction in mice receiving HDI of pKT2/CLP-AKT, pT/Caggs-NRASV12 and pCMV(CAT)T7-SB100. **a** Livers harvested from the mice at indicated time points after HDI. Yellow arrows indicate white spots or nodules on the liver. Hematoxylin-and-eosin (H&E) staining of liver sections from **b** mice at day 58 post HDI with DPBS (ctrl), **c** mice at day 30, **d** mice at day 45, or **e** mice at day 58 post HDI with DPBS containing the three plasmids. Yellow arrows in (**c**) and (**d**) indicated fatty change regions. The yellow arrow in **e** indicated mononuclear infiltration near the stroma; T: tumor region, L: adjacent liver tissue; Scale bars, 1000 or 100 μm

We first examined the capability and kinetics of activated Akt and N-Ras in HCC induction in C57BL/6 mice (Fig. 1b). We started to observe pinpointed white spots on the liver of a few injected C57BL/6j mice at day 30 post HDI with the three plasmids encoding myr-Akt1, N-RasV12, and sleeping beauty transposase. These pinpointed white spots on the liver were observed in nearly all of the injected mice by day 45 (Fig. 2a, yellow arrow). Some livers at day 45 and day 58, and all the livers by day 75 were covered with small nodules (Fig. 2a and data not shown). Histological examination of the liver tissues at day 30 post HDI revealed that most of the liver tissues were still normal except for the appearance of clusters of adipocyte-like cells in certain areas of the liver (Fig. 2b, c, yellow arrow). Eighty percent and 100% of the injected mice at day 45 and day 58 post HDI, respectively, had already developed HCC in the liver. Cytoplasmic basophilia and a high nuclear-to- cytoplasmic ratio were observed in cancer cells in tumor nodules. Fatty changes and ballooning changes of the tumor cells were abundant in the tumor tissues (Fig. 2d, e). Moreover, the tumor tissues had abundant mononuclear cells infiltrations and increased stroma cells especially at the edge of the tumor nodules (Fig. 2e, yellow arrow).

### Cellular composition in fatty change regions and late tumor tissues of the Akt1/N-Ras-induced HCC mouse model

**Fig. 3:**
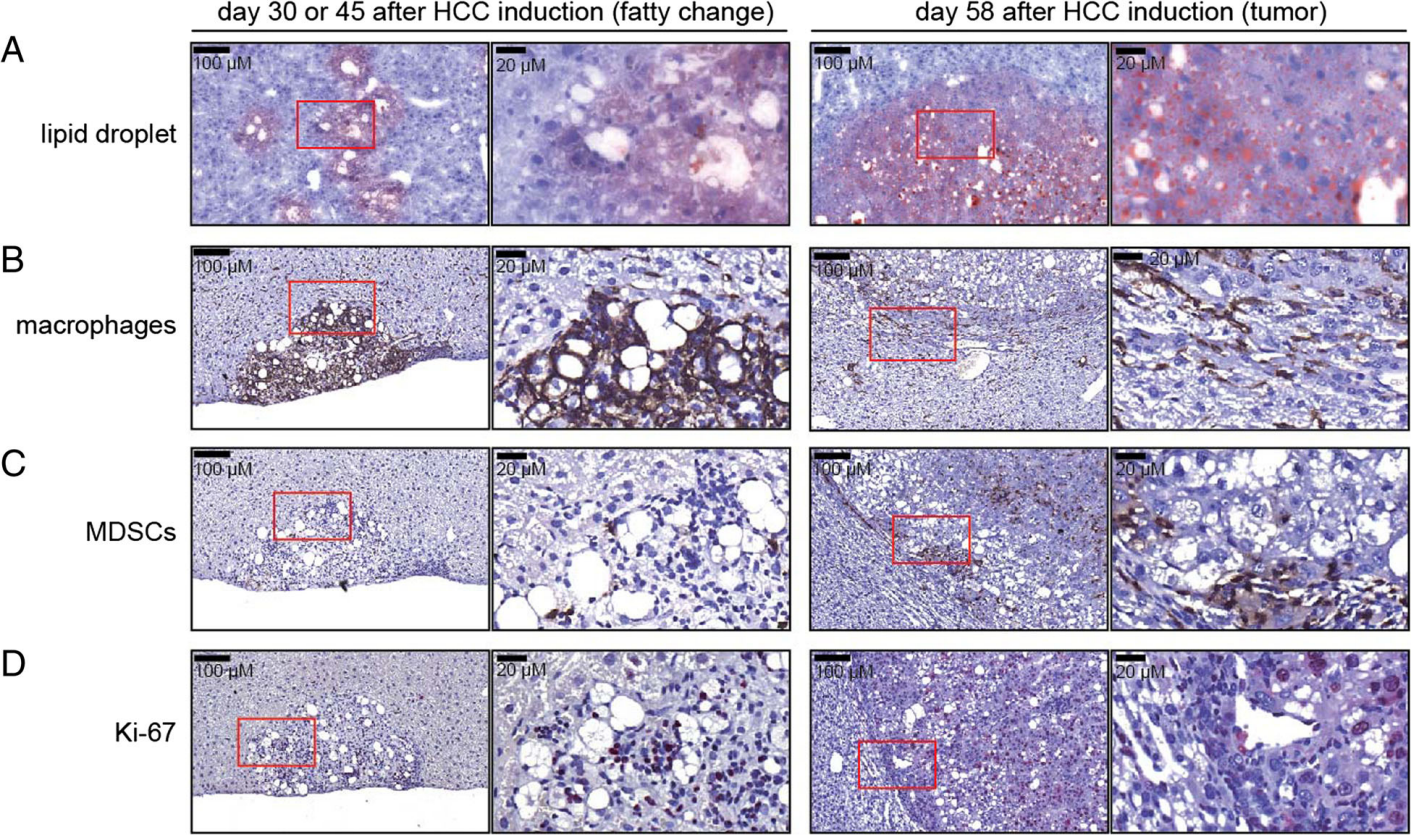
Immunohistochemical analysis of indicated proteins in the liver or tumor tissues of mice from Fig. 2. Tissues were collected at day 30 or 45 (fatty change) or day 58 (tumor) post HDI. **a** Oil red O, **b** F4/80, **c** Gr-1, and **d** Ki-67 staining of the liver/tumor tissues were shown. Scale bars, 100 or 20 μm

This spontaneous HCC model provided a good opportunity to reveal early immunologically cellular events during HCC progression. Activation of the Akt pathway can induce lipogenesis in hepatocytes, which subsequently induces carcinogenesis [9]. Marked lipid accumulation in the liver tissue from mice at day 30 or 45, and in tumor tissue at day 58 was confirmed via oil-red staining (Fig. 3a). We then profiled immune cells in early tissues with fatty changes and in late tumor tissues after HCC induction and observed that the majority of the immune cells attracted to the fatty change tissue were F4/80^+^ macrophages rather than Gr-1^+^ MDSCs (Fig. 3b, c). The infiltration of MDSCs increased in both the peritumoral and tumoral regions during HCC development (Fig. 3c, day 58). In the fatty change tissues, the major proliferative cells were mononuclear cells, most likely to be immune cells, but not hepatocytes; however, there were many Ki-67-positive cancer cells detected in the tumor region at day 58 after HCC induction (Fig. 3d).

We detected clustered CD4^+^ T cells, CD19^+^ B cells, and scattered CD8^+^ T cells in the fatty change tissues and late tumor tissues (see Additional file 2: Figure S1A-C). A few myofibroblasts expressing alpha-smooth muscle actin (α-SMA) were found in the fatty change tissues and they also accumulated in late tumor tissues; they were especially abundant in the peritumoral region (see Additional file 2: Figure S1D). CD31^+^ endothelial cells were not abundant in fatty change tissues but increased during HCC progression. The blood vessels in the tumor region showed irregularity and abnormality in morphology (see Additional file 2: Figure S1E). Our data suggest that during the onset of steatosis-related HCC, immune cells, especially macrophages and lymphocytes, infiltrated the fatty change tissues, which probably contributed to the later establishment of tumoral vasculature and proliferation of cancer stem cells. Moreover, the appearance of clustered CD4^+^ and CD19^+^ B cells in the stroma surrounding the tumor region suggests the emergence of ectopic lymphoid structures (ELSs) during HCC development in this model, similar to the findings in human HCC specimens and other HCC mouse models [16].

### Characterization of the Akt1/N-Ras-induced HCC mouse model with expression of luciferase and surrogate Ags

**Fig. 4:**
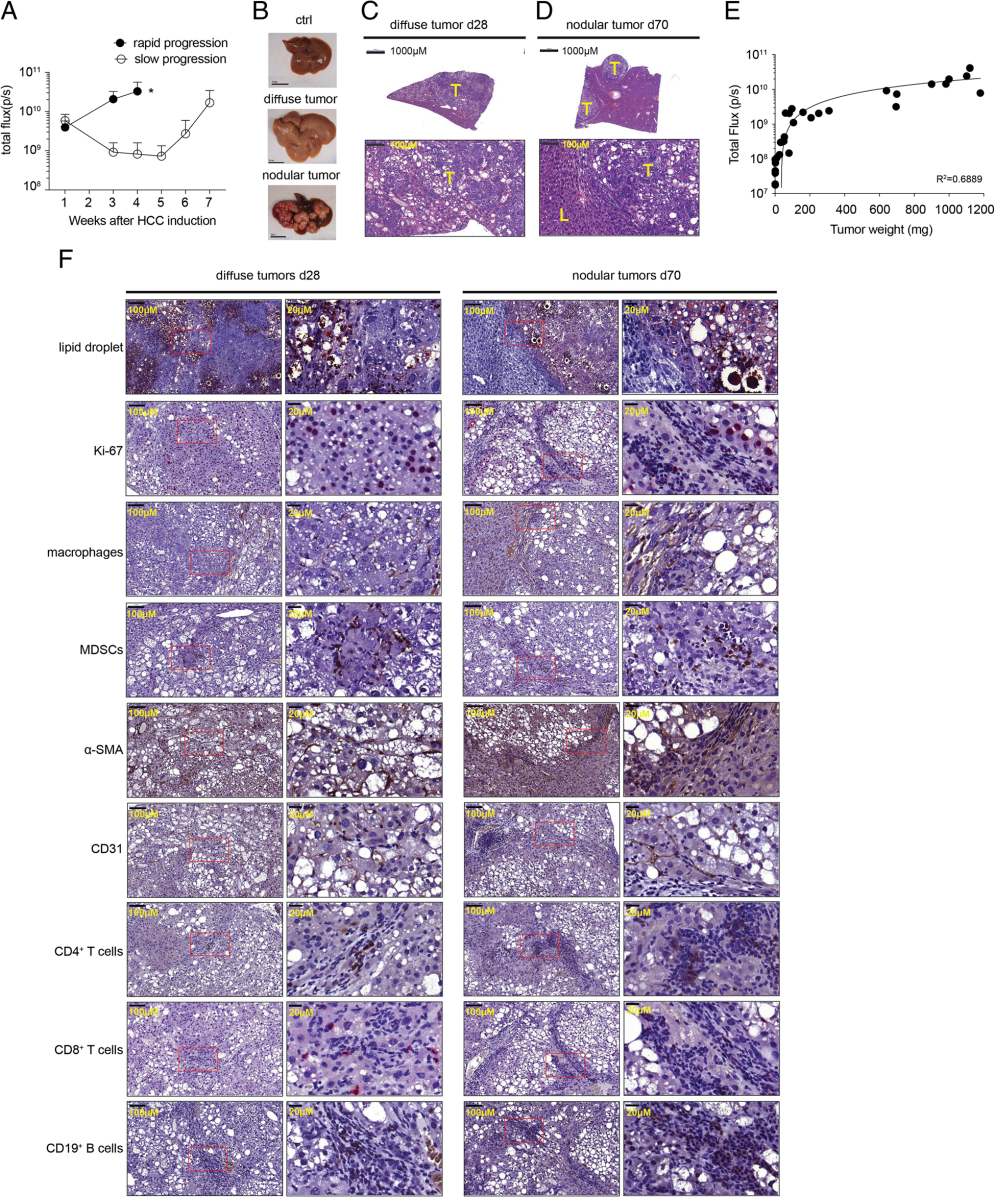
Both diffuse and nodular tumors were induced in C57BL/6j mice by HDI with pKT2/CLP-AKT-Ags-LUC, pT/Caggs-NRASV12 and pCMV(CAT)T7- SB100. **a** Bioluminescence of mice at indicated time points post HDI. Two different patterns, rapid and slow progression, of bioluminescence activity were shown. *The humane endpoint for tumor-bearing mice. **b** Representative livers harvested from the mice with rapid progression (d28) or slow progression (d70) after HDI. H&E staining of **c** the liver section from a representative mouse developing diffuse liver tumors at day 28 post HDI and **d** the liver section from one representative mouse developing nodular liver tumors at day 70 post HDI. T: tumor region, L: adjacent liver tissue; Scale bars, 1000 or 100 μm. **e** The correlation of the size of nodular tumors and their bioluminescence by IVIS (*n* = 29). **f** Staining of lipid, Ki-67, F4/80, Gr-1, α-SMA, CD31, CD4, CD8, CD19 of the diffuse and nodular tumor tissues were shown. Scale bars, 100 or 20 μm

We then examined the tumor growth and luciferase expression in C57BL/6 mice receiving HDI with the plasmids pKT2/CLP-AKT-OVA-HBc-HBs-LUC, pT/Caggs-NRASV12, and pCMV(CAT)T7-SB100 and found two different patterns of tumor growth in the injected mice. We expected that after HDI, the plasmids would transiently exist in hepatocytes as episomal forms and we did observe moderate luciferase activity in the first week after HDI (Fig. 4a). We then observed a decline in luciferase activity in these injected mice from the 1st week to the 5th week, probably due to the clearance of some hepatocytes harboring plasmids due to immune responses. An increase in luciferase activity was observed from the 5th week onwards and reached 10^10^ photons/second by the 7th week (Fig. 4a). The increase in luciferase activity is a reflection of tumor growth, resulting from transformation of the hepatocytes due to integration of ORFs of AKT-OVA-HBc-HBs-LUC and NRASV12. Interestingly, we observed that 1/3 of the mice receiving the three plasmids did not undergo the decline phase and their luciferase activity increased rapidly, reaching 10^10^ photons/second at the 3rd week (Fig. 4a). The liver of mice showing a rapid increase in luciferase looked pale and rough (Fig. 4b, middle panel), and appeared to be occupied by diffuse tumor infiltrations. The average weight of the liver with diffuse tumors was 3.968 ± 0.5721 g whereas that of the liver with nodular tumors was 1.823 ± 0.1531 g in the 6th week after induction. Histological examination revealed that the nodular tumors had clear boundaries between tumor and adjacent liver tissues, whereas the diffuse tumors invaded the normal liver tissues without a clear boundary between each other (Fig. 4c, d). We then measured the weight of the nodular tumors from each tumor-bearing mouse and correlated them with the total flux of the mice before being sacrificed, as measured using IVIS, and found a strong correlation between tumor weight and the luciferase activity. However, the correlation was lower when the total flux was higher than 10^10^ photons/seconds (Fig. 4e). Marked fatty change with a high level of lipid accumulation in the tissues of diffuse infiltrating tumors was observed and there were a lot of Ki-67-positive cells detected in the tumor tissues. Macrophages, MDSCs, and activated myofibroblasts were also abundant in the diffuse infiltrating tumors (Fig. 4f). There were CD31^+^ cells detected in the diffuse infiltrating tumors but less abundant in comparison with nodular tumors (Fig. 4f). Lymphocytes including CD4^+^ and CD8^+^ T cells and CD19^+^ B cells were also detected in the diffuse infiltrating tumors; however, they were scattered rather than clustered in the tumor tissues, differing from those in nodular tumors (Fig. 4f).

### Existence of TAA-specific CD8^+^ T cells in the Akt1/N-Ras-induced HCC mouse model

**Fig. 5:**
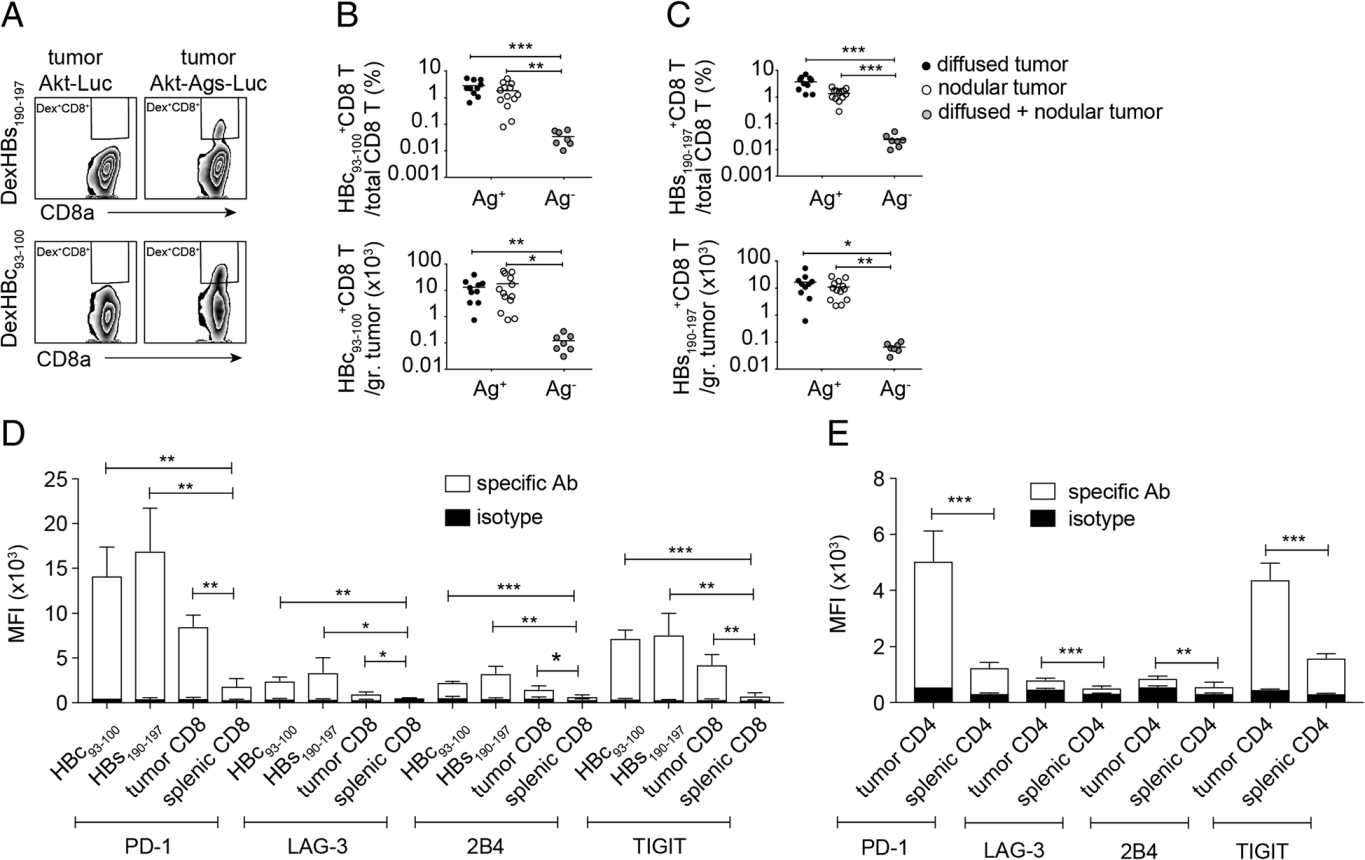
TAA-specific CD8^+^ T cells were detected in Akt1/N-Ras/Ag-induced HCC-bearing mice, however showed an exhausted-like phenotype in TME. **a** Gating for flow cytometric analysis of HBs_190–197_ or HBc_93–100_-specific CD8^+^ T cells among tumor-associated CD8^+^ T cells of mice receiving HDI of pKT2/CLP-AKT-LUC or pKT2/CLP-AKT-Ags-LUC, respectively, together with pT/Caggs-NRASV12 and pCMV(CAT)T7-SB100. The Percentage (top panel) and the absolute cell number (bottom panel) of **b** HBc_93–100_-specific CD8^+^ T cells and **c** HBs_190–197_-specific CD8^+^ T cells in nodular and diffused tumors expressing Ags (Ag+) or not expressing Ags (Ag-). Mean fluorescence intensity (MFI) of expression levels of PD-1, LAG-3, 2B4, and TIGIT on **d** intra-tumoral HBc_93–100_-specific CD8^+^ T cells, HBs_190–197_-specific CD8^+^ T cells, intra-tumoral total CD8^+^ T cells, and splenic total CD8^+^ T cells and on **e** intra-tumoral total CD4^+^ T cells and splenic total CD4^+^ T cells (*n* = 3–7 mice per group). *P < 0.05, **P < 0.01 and ***P < 0.001 (unpaired Student’s *t*-test)

A CD8^+^ T-cell response is critical for a HCC mouse model to be useful in drug screening and evaluation of therapeutic strategies, because CD8^+^ T cells are critical to tumor growth suppression and prevention of tumor recurrence. We collected tumor infiltrating leukocytes (TILs) from tumor tissues of mice receiving HDI with plasmids encoding AKT-OVA-HBc-HBs-LUC, NRASV12, and transposase, or receiving plasmids encoding AKT-LUC, NRASV12, and transposase. We then quantified the number of HBc_93–100_-, HBs-_190-197_-, and OVA_257–264_-specific CD8^+^ T cells using flow cytometric analysis. All populations of HBs-_190-197_-, HBc_93–100_-, and OVA_257–264_-specific CD8^+^ T cells were detected in the tumors of mice receiving the AKT-OVA-HBc-HBs-LUC plasmid (Ag^+^) but not in the tumors of mice receiving the AKT-LUC plasmid (Ag^−^) (Fig. 5a-c, see Additional file 3: Figure S2A-B), indicating the expression of these surrogate tumor Ags in the cancer cells. However, the appearance of TAA-specific CD8^+^ T cells did not control tumor growth and more than 90% of the injected mice developed HCC (Fig. 4a). We therefore analyzed the status of T-cell exhaustion of these TAA-specific CD8^+^ T cells and found that intratumoral HBs-_190-197_-, HBc_93–100_-, and OVA_257–264_-specific CD8^+^ T cells expressed relatively high levels of PD-1, LAG-3, 2B4, and TIGIT on their surface in comparison with those on total splenic CD8^+^ T cells (Fig. 5d, see Additional file 3: Figure S2C). High percentages of the TAA-specific CTLs expressed these immune checkpoints (see Additional file 3: Figure S2D-G, see Additional file 4: Figure S3A-D). The total intratumoral CD8^+^ T and CD4^+^ T cells also expressed higher levels of immune checkpoints on their surface when compared with total splenic CD8^+^ and CD4^+^ T cells, suggesting that the TME induces T-cell exhaustion (Fig. 5d, e, see Additional file 4: Figure S3A-H). Most of the intratumoral PD-1^hi^CD4^+^ and PD-1^hi^CD8^+^ T cells proliferated less and failed to produce cytokines in comparison with the PD-1^low/−^ cell population in response to in-vitro plate-bound anti-CD3/anti-CD28 re-stimulation (see Additional file 5: Figure S4A-E). These data clearly demonstrated that tumor-specific CTLs were indeed induced during HCC development; however, the Akt1/N-Ras induced HCC model showed strong immunosuppression in the TME and drove dysfunction of both CD4^+^ and CD8^+^ T cells, which may have been one of the reasons for the rapid tumor progression. However, transcriptional profiling should be performed to further reveal the exhausted status of these tumor infiltrating lymphocytes.

### Adoptively transferred TAA-specific CTLs were suppressed and failed to control tumor progression

**Fig. 6:**
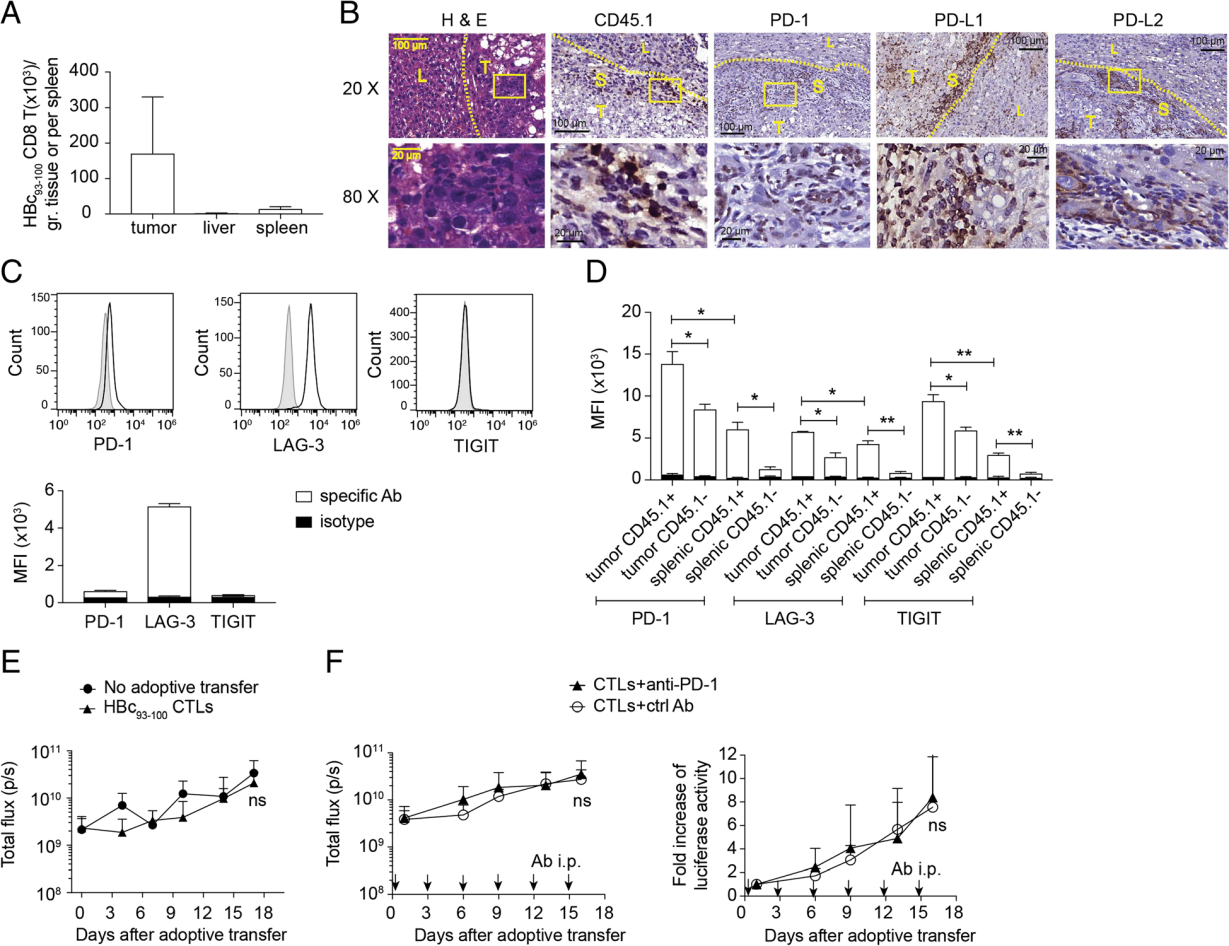
Adoptively transferred tumor-Ag-specific CD8^+^ T cells underwent T-cell exhaustion in tumor microenvironment. **a** Cell numbers of adoptively transferred HBc_93–100_-specific TCR tg CD8^+^ T cells in the tumors, spleens and livers of HCC-bearing mice at day 11 post adoptive transfer. **b** H&E staining and immunohistochemical staining of liver/tumor sections from (**a**) showed the tissue distribution of adoptively transferred tumor Ag-specific CD8^+^ T cells (CD45.1^+^; brown signals) and expression of PD-1, PD-L1 and PD-L2 in TME. T: tumor region, L: adjacent liver tissue, S: stroma; Expression of PD-1, LAG-3, and TIGIT on **c** in vitro pre-activated HBc_93–100_-specific CD8^+^ T cells at day 3 post activation by ant-CD3/anti-CD28 beads and on **d** intra-tumoral adoptively transferred tumor Ag-specific CD45.1^+^ CD8^+^ T cells, endogenous CD45.1^−^ CD8^+^ T cells, splenic adoptively transferred tumor Ag-specific CD45.1^+^ CD8^+^ T cells, and splenic endogenous CD45.1^−^ CD8^+^ T cells from the mice in (**a**). In vivo bioluminescence of HCC-bearing mice **e** receiving 2 × 10^5^ activated HBc_93–100_-specific CD8^+^ T cells or no adoptive transfer (*n* = 5 per group) or **f** receiving 3 × 10^5^ activated HBc_93–100_-specific CD8^+^ T cells together with anti-PD-1 blocking Ab or isotype control Ab (*n* = 8–9 per group). Arrows indicated the time points of Ab administration. ns, not significant; *P < 0.05, **P < 0.01 and ***P < 0.001 (unpaired Student’s *t*-test)

Adoptively transferred in-vitro activated CD45.1^+^ HBc_93–100_- specific CTLs from HBc_93–100_ TCR tg mice were found to be mainly located in tumor stromal regions (Fig. 6a-b). Numerous PD-1^+^ cells as well as PD-L1^+^ and PD-L2^+^ cells were found in the stroma or tumor mass (Fig. 6b). The CTLs, at the time of adoptive transfer, expressed nearly no PD-1 or TIGIT but high level of LAG-3 (Fig. 6c) and then expressed higher levels of PD-1 and TIGIT when entering HCC TME (Fig. 6c-d, see Additional file 6: Figure S5A-C). These adoptively transferred CTLs failed to control tumor progression (Fig. 6e) even in combination with anti-PD-1 treatment (Fig. 6f), suggesting that there are regulatory cues other than PD-1 signaling contributing to the unresponsiveness of these TAA-specific CTLs. More investigations are needed to further dissect the roles of each immune checkpoint in regulation of functions and survival of endogenous and adoptively transferred TAA-specific CTLs in this model.

### Accumulation of immunosuppressive cell populations in Akt1/N-Ras-induced HCC-bearing mice

**Fig. 7:**
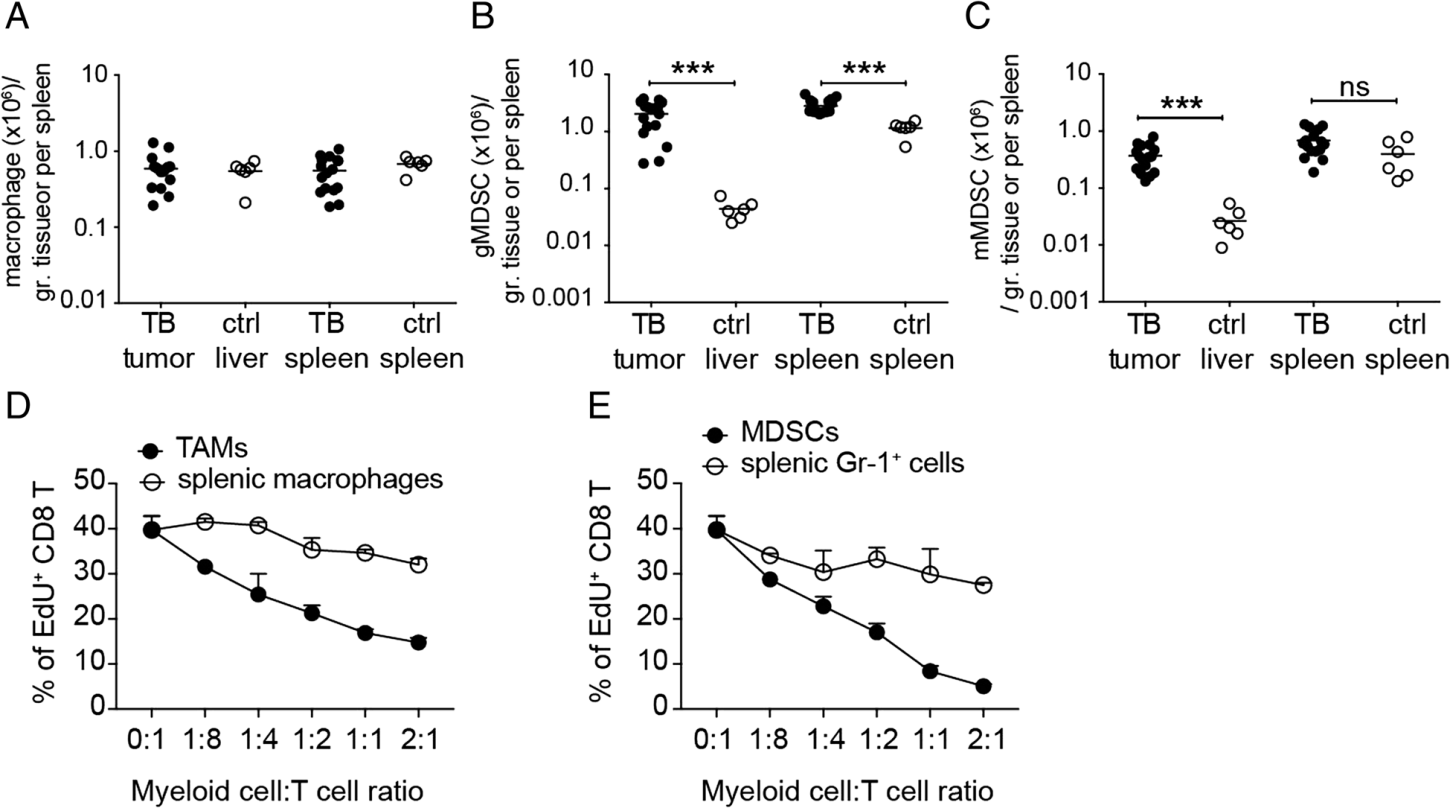
Profile of infiltrating myeloid cells and their immunosuppression. Cell numbers of **a** macrophages, **b** granulocytic MDSCs (gMDSCs) and **c** monocytic (mMDSCs) in tumor tissues and spleens of tumor-bearing mice (TB) as well as in liver tissues and spleens of control normal C57BL/6j mice (ctrl) were shown. Proliferation of HBc_93–100_-specific CD8^+^ T cells (8 × 10^4^/well) co-cultured with different numbers of **d** FACSorted tumor-associated macrophages (TAMs) and FACSorted splenic macrophages, respectively, or **e** FACSorted intra-tumoral myeloid-derived suppressor cells (MDSCs), and FACSorted splenic Gr-1^+^ myeloid cells, respectively. The proliferation of T cells was determined by EdU incorporation assay and the percentage of EdU^+^ CD8^+^ T cells among total CD8^+^ T cells was shown. ns, not significant; ***P < 0.001 (unpaired Student’s *t*-test)

Myeloid cells including TAMs and MDSCs are critical for tumor progression. We therefore analyzed the cell number of myeloid cells in the tumor and in the spleen of tumor-bearing mice and compared them with the cell number in normal control mice. Macrophages were quite abundant in tumor tissues, nearly as enriched as Kupffer cells in liver tissues of normal mice (Fig. 7a). Splenic macrophages did not increase in tumor-bearing mice (Fig. 7a) whereas granulocytic MDCSs increased in the spleens of tumor-bearing mice (Fig. 7b). Both granulocytic and monocytic MDCSs were enriched in the tumor tissues in comparison to the normal liver tissues (Fig. 7b, c). When activated CTLs were re-stimulated and co-cultured with FACSorted macrophages or MDSCs from tumor tissues, their proliferation was severely affected by the two types of myeloid cells. The tumor-associated MDSCs inhibited T-cell proliferation at a very low myeloid/T ratio, suggesting a stronger immunosuppressive capability of MDSCs than that of TAMs (Fig. 7d, e). In addition to the myeloid cell population, there were also regulatory T cells (Treg) found in the HCC TCM, which may also have contributed to the immunosuppression in the TME (see Additional file 7: Figure S6A-B). The percentage of intratumoral Treg among the CD4^+^ T-cell population was similar to that in the splenic compartment. Interestingly, the ratio of CD8^+^ T versus CD4^+^ T cells in the tumor was higher than in the spleen, implying that the HCC TME preferentially attracted CD8^+^ T cells but suppressed their effector functions efficiently to ensure the growth of cancer cells [17]. The spontaneous HCC mouse model we developed could serve as a useful tool to address the kinetics and mechanisms of immune cell migration and cell-cell interaction during HCC progression, providing useful information for development of therapeutic strategies for HCC treatment.

## Discussion

In the present study, we found that macrophages, earlier than MDSCs or lymphocytes, entered the fatty change tissues in the liver upon HCC induction. Studies in non-alcoholic fatty liver disease (NAFLD) and non-alcoholic steatohepatitis (NASH) have shown that steatosis or accumulated free fatty acids caused death of hepatocytes [18]. The release of danger-associated molecular pattern (DAMPs) or apoptotic hepatocytes could activate non-parenchymal cell populations e.g. Kupffer cells and hepatic stellate cells and drive them to produce cytokines, chemokines, and reactive oxygen species (ROS) in the local microenvironment to drive repair processes, but could possibly trigger recruitment and differentiation of immune cells into liver tissue and induce chronic inflammation [19–21]. NFκ-B and STAT3 signaling pathways were shown to be the key players connecting chronic inflammation and hepatocarcinogenesis [21].

It is very likely that activation of the Akt pathway in hepatocytes in this model recapitulates aberrant lipid metabolism during NAFLD, resulting in accumulation of free fatty acids and death of hepatocytes, subsequently activating liver resident cells and immigratory immune cells to initiate chronic hepatitis and further liver cancer. The macrophages in the steatosis and dysplasia lesions found after activation of the Akt pathway in this model are most likely to be monocytic in origin, recruited to the inflamed liver through the CCR2-CCL2 axis, CCL1-CCR8 axis, interaction between ICAM-1 and CD44, or sphingosine 1-phosphate receptor 2/3-mediated trafficking. Although it has been shown that Akt and N-Ras co-activation could rapidly increase proliferation and angiogenesis of HCC cells via mTORC1, FOXM1/SKP2, and c-Myc pathways [7], we still could not exclude that early recruitment of the macrophages into fatty change lesions of the Akt1/N-Ras-induced HCC contributes to the HCC progression. The blockade of monocytes into fatty change lesion using a pharmacological CCR2 antagonist in this model may assist to tackle the above question [22]. It has been shown that hepatocarcinogenesis induced by AKT/c-Met activation was totally abolished in conditional fatty acid synthase (FASN) knockout mice, suggesting an important role of lipogenesis in HCC progression in this model. Whether the lipogenesis per se or the chronic inflammation induced by the release of free fatty acids due to the lipogenesis contributes to the HCC progression in the Akt1/N-Ras-induced HCC mouse model can be further addressed using FASN inhibitors, ROS inhibitors, or genetically modified mouse strains. The later appearance of MDSCs in the tumor excludes a major role of MDSCs in the initiation of hepatocarcinogenesis in this model. MDSCs have been shown to play important roles in the suppression of effector T cells and in the promotion of Treg response in various types of cancer patients including HCC patients [23–25].

Human HCC can present in several different subtypes grossly including nodular, massive, and diffuse/infiltrative tumors [26, 27]. In this Akt1/N-Ras-induced mouse model, we observed two different subtypes of HCC induced after HDI of the same three plasmids. In diffuse HCC, the cancer cells develop at the very beginning after induction and proliferate vigorously, which was evident from the high percentage of tumor cells expressing Ki-67. There were very few Ki-67-positive hepatocytes in fatty change lesions. It took a longer time to develop nodular HCC from fatty change lesions after induction. One of the possible and reasonable explanations for this phenomenon is that the in vivo transduction rate of HDI may vary in individual mice. The number of initially transduced hepatocytes after HDI may be more in the diffuse HCC group than in nodular HCC group, resulting in rapid disease progression in the diffuse group. It has been shown in the HBV mouse model that a high dose of plasmid in HDI would induce intracellular innate immune responses and subsequently affect the persistence rate of HBV and generation of an adaptive immune response against HBV [28]. Therefore, the plasmid doses in HDI may affect the integration efficiency of the sleeping beauty transposon, which could lead to differential tumor progression in this Akt1/N-Ras HCC mouse model. Liver regeneration was found to enhance growth or recurrence of HCC [29, 30]. Because HDI per se induces hepatocytes damage during the first 2 days after injection, the HDI-associated liver damage and subsequent regeneration may accelerate tumorigenesis of Akt1/N-Ras-transformed hepatocytes. The degree of hepatocyte damage induced by HDI may vary in each mouse, resulting in both diffuse and nodular types of HCC in this model.

There were fewer stroma cells found around diffuse tumors, probably due to the rapid disease progression in the diffuse group, resulting in insufficient time for the development of chronic inflammation. Conversely, we first observed fatty changes in the liver of mice receiving HDI and simultaneously, macrophages were recruited to the dysplasia area and were probably activated by the dead hepatocytes to release cytokines and chemokines, which would mediate the recruitment and activation of stroma cells and immune cells to further support the development of nodular carcinoma. This process mimics the inflammatory hepatocarcinogenesis induced by NASH [18, 20]. The two subtypes of HCCs developed in the Akt1/N-Ras HCC mouse model could be easily distinguished based on the pattern of in vivo bioluminescence, and therefore are useful in studies characterizing differences in immunological features between nodular HCCs and diffuse HCCs and also for development of therapeutic strategies for treatment of the two subtypes of HCCs.

A recent study on the immune landscape of cancers showed that HCC belongs to the lymphocyte-deficient subtype with high M2 macrophage and suppressive Th1 responses [31]. We indeed observed a large amount of suppressive macrophages infiltrating in the Akt1/N-Ras-induced HCC tissue and impairment of CTL responses against the tumor. The endogenous or adoptively transferred TAA-specific CD8^+^ T cells were retained mainly in the stroma and showed exhausted-like phenotypes. Our finding suggests that overcoming the immunosuppressive TME of HCC is critical for the development of immunotherapy targeting HCC. Recently, immune checkpoint inhibitors and T-cell based therapy are being investigated vigorously for the treatment of various cancers. Treatment of cancer patients with anti-PD-1 has achieved 10–30% responsive rate and around 70% disease control rate in HCC patients [32]. The expression of immune checkpoints other than PD-1 on CTLs in the HCC TME was found in our study and in previous reports [33–37]. The expression of surrogate tumor Ags and a convenient method for monitoring the tumor progression and for detection and analysis of TAA-specific T-cell responses, as illustrated in the present study, make the Akt1/N-Ras induced HCC mouse model we developed a suitable preclinical model for examination of immune checkpoint inhibitors and cell-based immunotherapy for HCC treatment.

## Conclusions

We have generated and characterized a novel Akt1/N-Ras-induced HCC mouse model which enables researchers to monitor tumor growth non-invasively. The tumor progression in this HCC model is more rapid than in most of the chemically induced models and GEMs; based on measurement of luciferase activity using IVIS, tumors size could be estimated for grading tumor progression. The expression of surrogate tumor Ags in cancer cells in the model enables the researchers to elucidate crosstalk between TAA-specific T cells and stromal cells, and the underlying mechanisms governing immunosuppression in the HCC TME. We illustrated the induction of TAA-specific T-cell responses and T-cell exhaustion occurring in liver cancer in our study, suggesting that the Akt1/N-Ras-induced HCC mouse model we developed is a suitable preclinical model for examination of immune checkpoint inhibitors and cell-based immunotherapy for HCC treatment.

## Additional files

Additional file 1: **Table S1.** List of antibodies used in immunohistochemistry and in flow cytometry. (PDF 37 kb)

Additional file 2: **Figure S1.** Immune cell profiling in the liver or tumor tissues from mice receiving HDI of pKT2/CLP-AKT, pT/Caggs-NRASV12 and pCMV(CAT)T7-SB100. (PDF 627 kb)

Additional file 3: **Figure S2.** Characterization of OVA257-264-specific CTLs in HCC tumor microenvironment. (PDF 367 kb)

Additional file 4: **Figure S3.** The expression of immune checkpoints on CD8+ T cells and CD4 + T cells from mice receiving HDI of pKT2/CLP-AKT-Ags-LUC, pT/Caggs-NRASV12 and pCMV(CAT)T7-SB100. (PDF 313 kb)

Additional file 5: **Figure S4.** Proliferation and cytokine production of intra-tumoral and splenic CD8+ and CD4+ T cells after re-stimulation. (PDF 275 kb)

Additional file 6: **Figure S5.** The expression of immune checkpoints on adoptively transferred TAA-specific CD8^+^ T cells. (PDF 163 kb)

Additional file 7: **Figure S6.** Lymphocyte populations in HCC tumor microenvironment. (PDF 200 kb)

## Abbreviations

Ag: Antigen
DPBS: Dulbecco Phosphate Buffered Saline
HBcAg: Hepatitis B core antigen
HBsAg: Hepatitis B surface antigen
HCC: Hepatocellular carcinoma
HDI: Hydrodynamic injection
MDSC: Myeloid-derived suppressor cell
MHC: Major histocomplexicity
N-Ras: Neuroblastoma Ras
ORF: Open reading frame
OVA: Ovalbumin
TAA: Tumor-associated antigen
TAM: Tumor-associated macrophage
TCR: T cell receptor
tg: transgenic
TIL: Tumor infiltrating leukocytes
TME: Tumor microenvironment
Treg: Regulatory T cells
