# Supplementary material for: A novel spontaneous hepatocellular carcinoma mouse model for studying T-cell exhaustion in the tumor microenvironment
Source: J Immunother Cancer. 2018 Dec 7;6:144. doi: 10.1186/s40425-018-0462-3 (PMC6286542; doi:10.1186/s40425-018-0462-3)
Supplement: Supplementary file 1 — Table S1. List of antibodies used in immunohistochemistry and in flow cytometry. (PDF 37 kb) [file 40425_2018_462_MOESM1_ESM.pdf]

Table S1 List of antibodies used in immunohistochemistry

Antigen	Ab clone	Isotype	Vendor
F4/80	BM8	Rat IgG2a, κ	Biolegend, San Diego, USA
Gr-1	RB6-8C5	Rat IgG2b, κ	Biolegend
CD4	D7D2Z	Rabbit IgG	Cell Signaling Technology, Danvers, USA
CD8	D4W2Z	Rabbit IgG	Cell Signaling Technology
CD19	6OMP31	Rat IgG2a, κ	Thermo Fisher Scientific, Waltham, USA,
CD31	Polyclonal	Rabbit IgG	Abcam, Cambridge, UK
alpha-Smooth muscle actin (α-SMA)	Polyclonal	Rabbit IgG	Abcam
Ki-67	SP6	Rabbit IgG	Abcam
CD45.1	A20	Mouse IgG2a, κ	Thermo Fisher Scientific
PD-1	29F.1A12	Rat IgG2a, κ	Bio X cell, New Hampshire, USA
PD-L1	10F9G2	Rat IgG2b, κ	Biolegend
PD-L2	D7U8C	Rabbit IgG	Cell Signaling Technology

Table S2 List of antibodies used in flow cytometry

Antigen	Ab clone	Conjugation	Vendor
PD-1	J43	Fluorescein isothiocyanate (FITC)	Thermo Fisher Scientific
2B4	m2B4 (B6) 458.1	FITC	Biolegend
LAG-3	C9B7W	Brilliant Violet 421 (BV421)	Biolegend
TIGIT	1G9	BV421	Biolegend
CD4	RM4-5	Brilliant Violet 510 (BV510)	BD Biosciences, CA, USA
CD8α	53-6.7	Phycoerythrin (PE)	Biolegend
CD11b	M1/70	PE	Thermo Fisher Scientific
CD25	PC61	Allophycocyanin (APC)	Biolegend
CD45.1	A20	PerCp-Cy5.5	Biolegend
CD146	ME-9F1	Alexa Fluor 488	Biolegend
Gr-1	RB6-8C5	PerCp-Cy5.5	Biolegend
Ly6C	HK1.4	APC	Biolegend
Ly6G	1A8	PerCp-Cy5.5	Biolegend
F4/80	BM8	Alexa Fluor 700	Biolegend
MHC class II I-A/I-E	M5/114.15.2	eFluor 450	Biolegend
Foxp3	MF-14	BV421	Biolegend
NK1.1	PK136	PE/Cy7	Thermo Fisher Scientific
IL-2	JES6-5H4	PE/Cy7	BD Biosciences
IFNγ	XMG1.2	FITC	Biolegend

TNFα	MP6-XT22	PE	Thermo Fisher Scientific
LIVE/Dead fixable dye	-	Near-IR	Thermo Fisher Scientific
LIVE/Dead fixable dye	-	Far Red	Thermo Fisher Scientific
