# Supplementary material for: A novel spontaneous hepatocellular carcinoma mouse model for studying T-cell exhaustion in the tumor microenvironment
Source: J Immunother Cancer. 2018 Dec 7;6:144. doi: 10.1186/s40425-018-0462-3 (PMC6286542; doi:10.1186/s40425-018-0462-3)
Supplement: Supplementary file 2 — Figure S1. Immune cell profiling in the liver or tumor tissues from mice receiving HDI of pKT2/CLP-AKT, pT/Caggs-NRASV12 and pCMV(CAT)T7-SB100. (PDF 627 kb) [file 40425_2018_462_MOESM2_ESM.pdf]

Figure S1

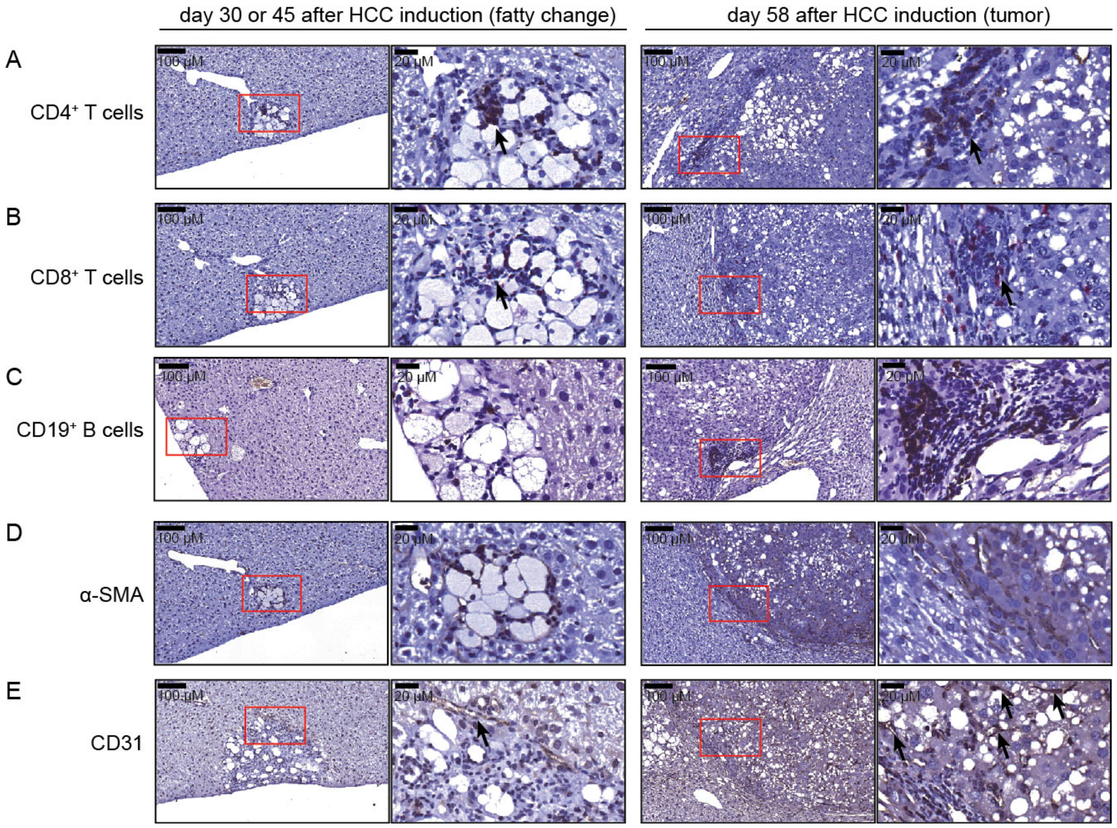

Figure S1 Immunohistochemical analysis of indicated proteins in the liver or tumor tissues from mice receiving HDI of pKT2/CLP-AKT, pT/Caggs-NRASV12 and pCMV(CAT)T7-SB100. Tissues were collected at day 30 or 45 (fatty change) or day 58 (tumor) post HDI. (**A**) CD4 staining, (**B**) CD8 staining, (**C**) CD19 staining, (**D**) α-SMA staining, and (**E**) CD31 staining of the liver/tumor tissues were shown. Scale bars, 100 or 20 μm
