# Supplementary material for: A novel spontaneous hepatocellular carcinoma mouse model for studying T-cell exhaustion in the tumor microenvironment
Source: J Immunother Cancer. 2018 Dec 7;6:144. doi: 10.1186/s40425-018-0462-3 (PMC6286542; doi:10.1186/s40425-018-0462-3)
Supplement: Supplementary file 3 — Figure S2. Characterization of OVA257-264-specific CTLs in HCC tumor microenvironment. (PDF 367 kb) [file 40425_2018_462_MOESM3_ESM.pdf]

Figure S2

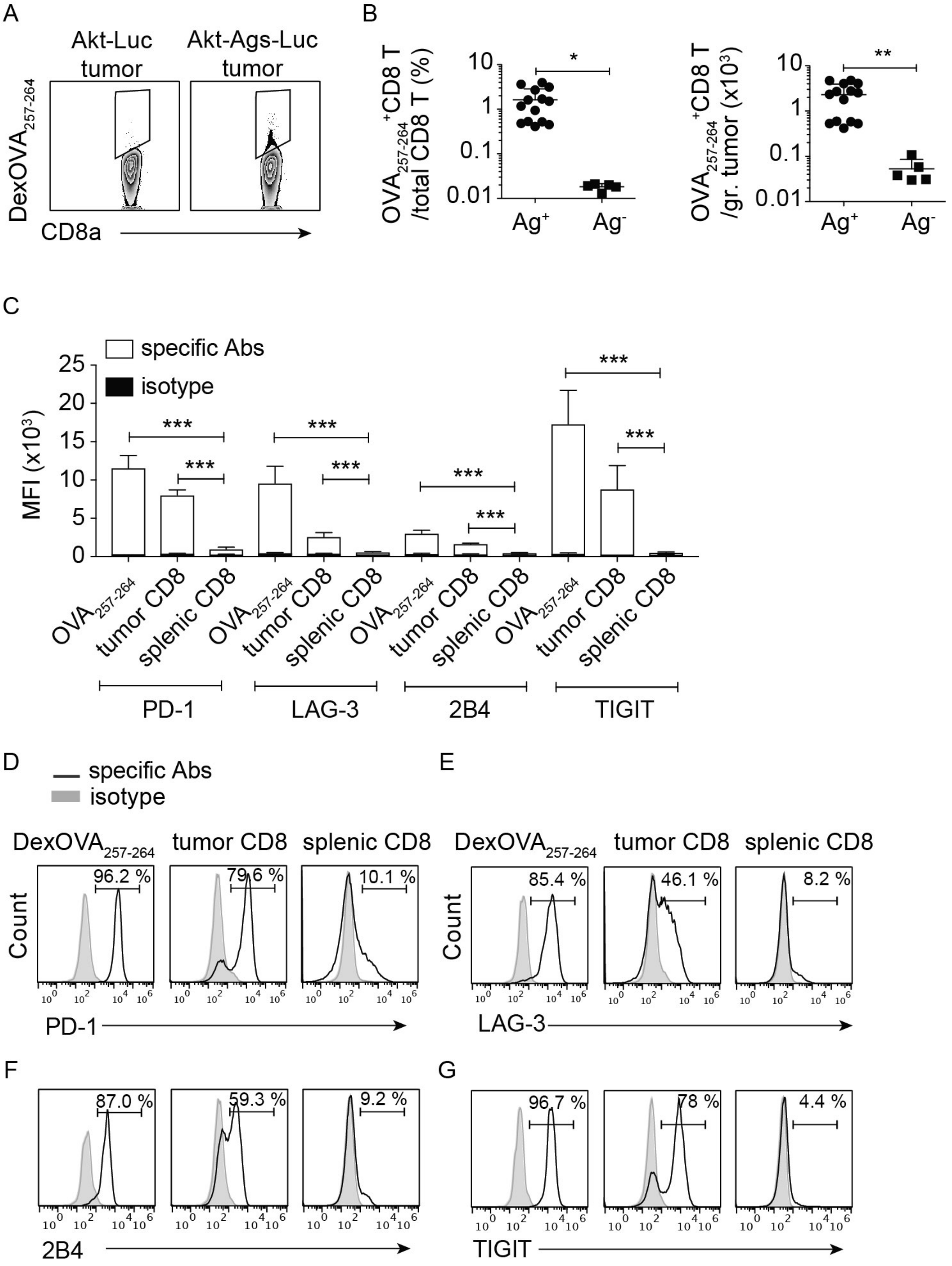

Figure S2 Tumor Ag-specific CD8$^+$ T cells were induced in Akt1/N-Ras/Ag-induced HCC-bearing mice, however underwent T-cell exhaustion in tumor microenvironment. (**A**) Gating for flow cytometric analysis of OVA$_{257-264}$-specific CD8$^+$ T cells among tumor-associated CD8$^+$ T cells of mice receiving HDI of pKT2/CLP-AKT-LUC or pKT2/CLP-AKT-Ags-LUC, respectively together with

pT/Caggs-NRASV12 and pCMV(CAT)T7-SB100. (**B**) The Percentage (left panel) and the absolute cell number (right panel) of $OVA_{257-264}$-specific $CD8^+$ T cells in tumors expressing Ags (Ag+) or not expressing Ags (Ag-). (n=5-14 mice per group). (**C**) Mean fluorescence intensity (MFI) of expression levels of PD-1, LAG-3, 2B4, and TIGIT on intra-tumoral $OVA_{257-264}$-specific $CD8^+$ T cells, intra-tumoral total $CD8^+$ T cells and splenic total $CD8^+$ T cells. (n=7 mice). Representative histograms for expression levels of (**D**) PD-1, (**E**) LAG-3, (**F**) 2B4, and (**G**) TIGIT on indicated cell populations. The average percentage of positive cells for each immune checkpoint staining was indicated in the upper right corner of the plot. *P < 0.05, **P < 0.01 and ***P < 0.001 (unpaired Student's *t*-test)
