# Supplementary material for: A novel spontaneous hepatocellular carcinoma mouse model for studying T-cell exhaustion in the tumor microenvironment
Source: J Immunother Cancer. 2018 Dec 7;6:144. doi: 10.1186/s40425-018-0462-3 (PMC6286542; doi:10.1186/s40425-018-0462-3)
Supplement: Supplementary file 4 — Figure S3. The expression of immune checkpoints on CD8+ T cells and CD4 + T cells from mice receiving HDI of pKT2/CLP-AKT-Ags-LUC, pT/Caggs-NRASV12 and pCMV(CAT)T7-SB100. (PDF 313 kb) [file 40425_2018_462_MOESM4_ESM.pdf]

Figure S3

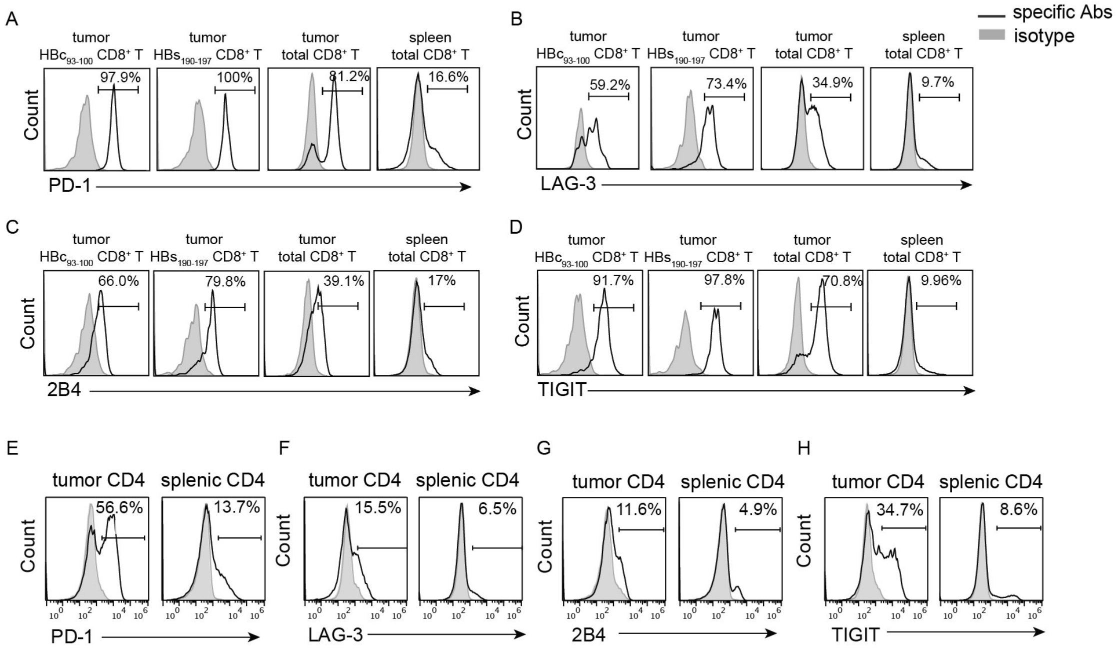

Figure S3 The expression of immune checkpoints on $CD8^+$ T cells and $CD4^+$ T cells from mice receiving HDI of pKT2/CLP-AKT-Ags-LUC, pT/Caggs-NRASV12 and pCMV(CAT)T7-SB100. Representative histograms for expression levels of (A) PD-1, (B) LAG-3, (C) 2B4, and (D) TIGIT on intra-tumoral HBc_{93-100}-specific $CD8^+$ T cells, $HBs_{190-197}$-specific $CD8^+$ T cells, intra-tumoral total $CD8^+$ T cells, and splenic total $CD8^+$ T cells were shown. (n=3 mice). Representative histograms for expression levels of (E) PD-1, (F) LAG-3, (G) 2B4, and (H) TIGIT on intra-tumoral and splenic $CD8^-CD4^+$ T cells were shown. (n=7 mice). The average percentage of positive cells for each immune checkpoint staining was indicated in the upper right corner of the plot.
