# Supplementary material for: A novel spontaneous hepatocellular carcinoma mouse model for studying T-cell exhaustion in the tumor microenvironment
Source: J Immunother Cancer. 2018 Dec 7;6:144. doi: 10.1186/s40425-018-0462-3 (PMC6286542; doi:10.1186/s40425-018-0462-3)
Supplement: Supplementary file 5 — Figure S4. Proliferation and cytokine production of intra-tumoral and splenic CD8+ and CD4+ T cells after re-stimulation. (PDF 275 kb) [file 40425_2018_462_MOESM5_ESM.pdf]

Figure S4

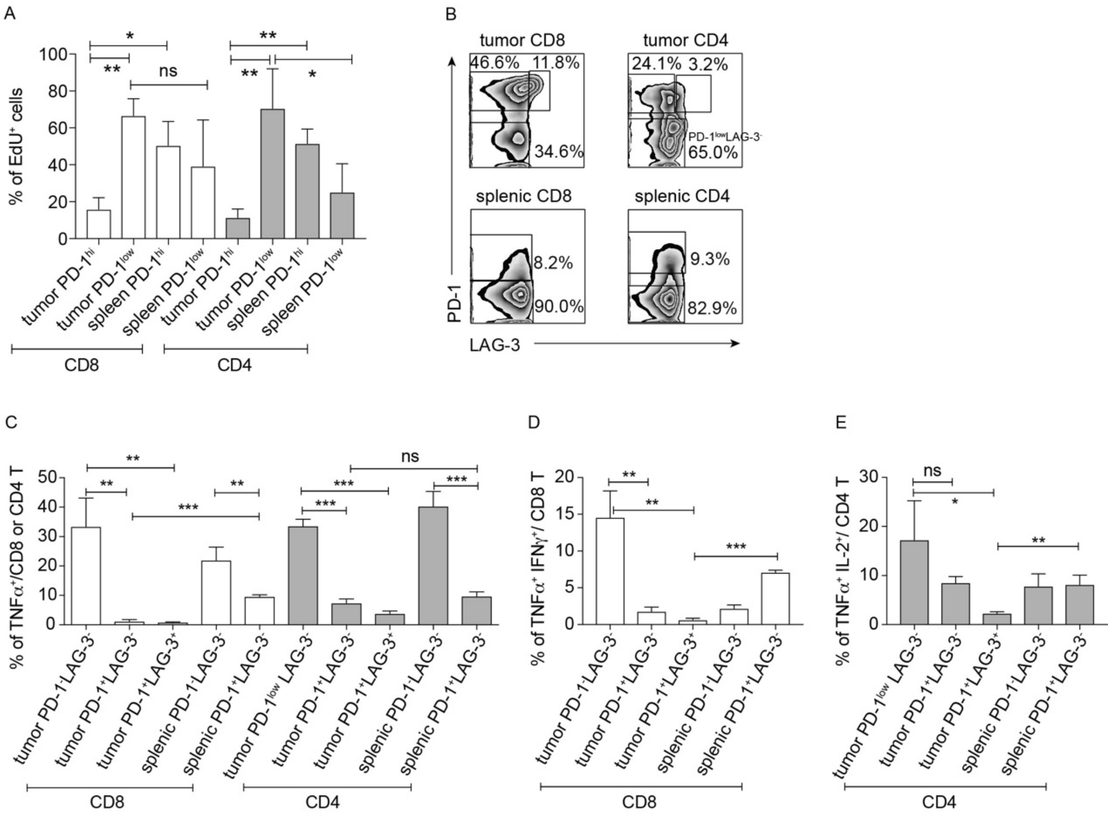

Figure S4 Proliferation and cytokine production of intra-tumoral and splenic CD8+ and CD4+ T cells after re-stimulation. (**A**) Proliferation of intra-tumoral PD-1^{hi}CD8^{+}, PD-1^{low}CD8^{+}, splenic PD-1^{hi}CD8^{+}, PD-1^{low}CD8^{+}, intra-tumoral PD-1^{hi}CD4^{+}, PD-1^{low}CD4^{+} T cells, splenic PD-1^{hi}CD4^{+}, and PD-1^{low}CD4^{+} T cells. The tumor infiltrating leukocytes and splenocytes were subjected to plate-bound anti-CD3/anti-CD28 activation for 54 hours. The proliferation of T cells was determined by EdU incorporation during the last 6-hours of activation and the percentage of EdU^{+} CD8^{+} T cells among indicated CD8^{+} or CD4^{+} T-cell populations was shown. (n=3 mice). (**B**) Gating for flow cytometric analysis of intracellular cytokines in indicated T-cell populations. The tumor infiltrating leukocytes and splenocytes were subjected to plate-bound anti-CD3/anti-CD28 activation for 8 hours and harvested for the staining of surface markers and intracellular cytokines, followed by flow cytometric analysis. Brefeldin A (3 μg/ml) and Monensin (2 μM) were added to the cell culture media during the last 5-hrs of activation. The percentages of (**C**) TNFα single positive cells, (**D**) TNFα and IFNγ double positive cells and (**E**) TNFα and IL-2 double positive cells among the indicated cell populations were shown. (n=3 mice). ns, not significant; *P < 0.05, **P < 0.01 and ***P < 0.001 (unpaired Student's *t*-test)
