# Supplementary material for: A novel spontaneous hepatocellular carcinoma mouse model for studying T-cell exhaustion in the tumor microenvironment
Source: J Immunother Cancer. 2018 Dec 7;6:144. doi: 10.1186/s40425-018-0462-3 (PMC6286542; doi:10.1186/s40425-018-0462-3)
Supplement: Supplementary file 6 — Figure S5. The expression of immune checkpoints on adoptively transferred TAA-specific CD8+ T cells. (PDF 163 kb) [file 40425_2018_462_MOESM6_ESM.pdf]

Figure S5

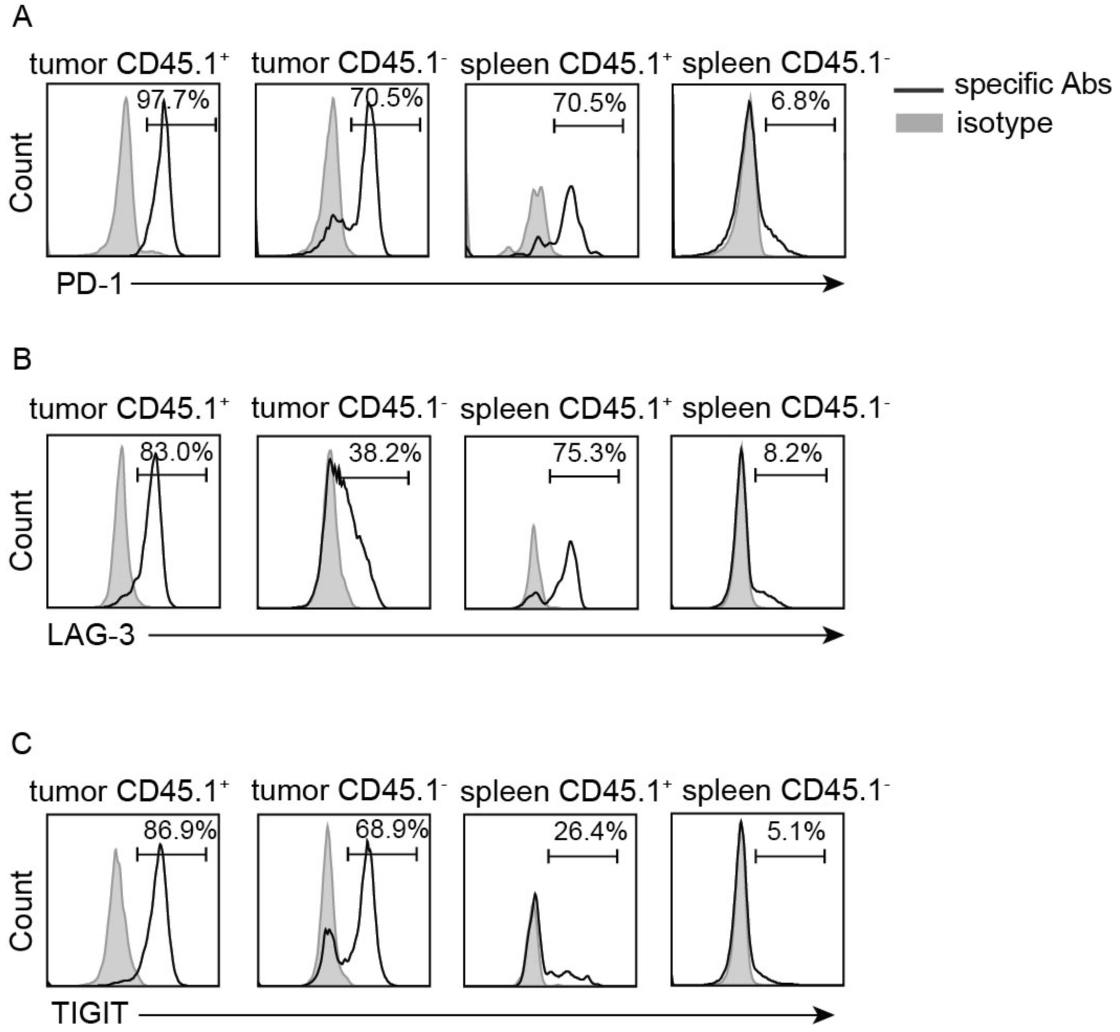

Figure S5 The expression of immune checkpoints on adoptively transferred TAA-specific $CD8^+$ T cells. Representative histograms for expression levels of (**A**) PD-1, (**B**) LAG-3, and (**C**) TIGIT on intra-tumoral adoptively transferred $CD45.1^+CD8^+$ T cells, $CD45.1^-CD8^+$ T cells (endogenous), splenic adoptively transferred $CD45.1^+CD8^+$ T cells, and splenic $CD45.1^-CD8^+$ T cells (endogenous). The average percentage of positive cells for each immune checkpoint staining was indicated in the upper right corner of the plot.
