# Supplementary material for: A novel spontaneous hepatocellular carcinoma mouse model for studying T-cell exhaustion in the tumor microenvironment
Source: J Immunother Cancer. 2018 Dec 7;6:144. doi: 10.1186/s40425-018-0462-3 (PMC6286542; doi:10.1186/s40425-018-0462-3)
Supplement: Supplementary file 7 — Figure S6. Lymphocyte populations in HCC tumor microenvironment. (PDF 200 kb) [file 40425_2018_462_MOESM7_ESM.pdf]

Figure S6

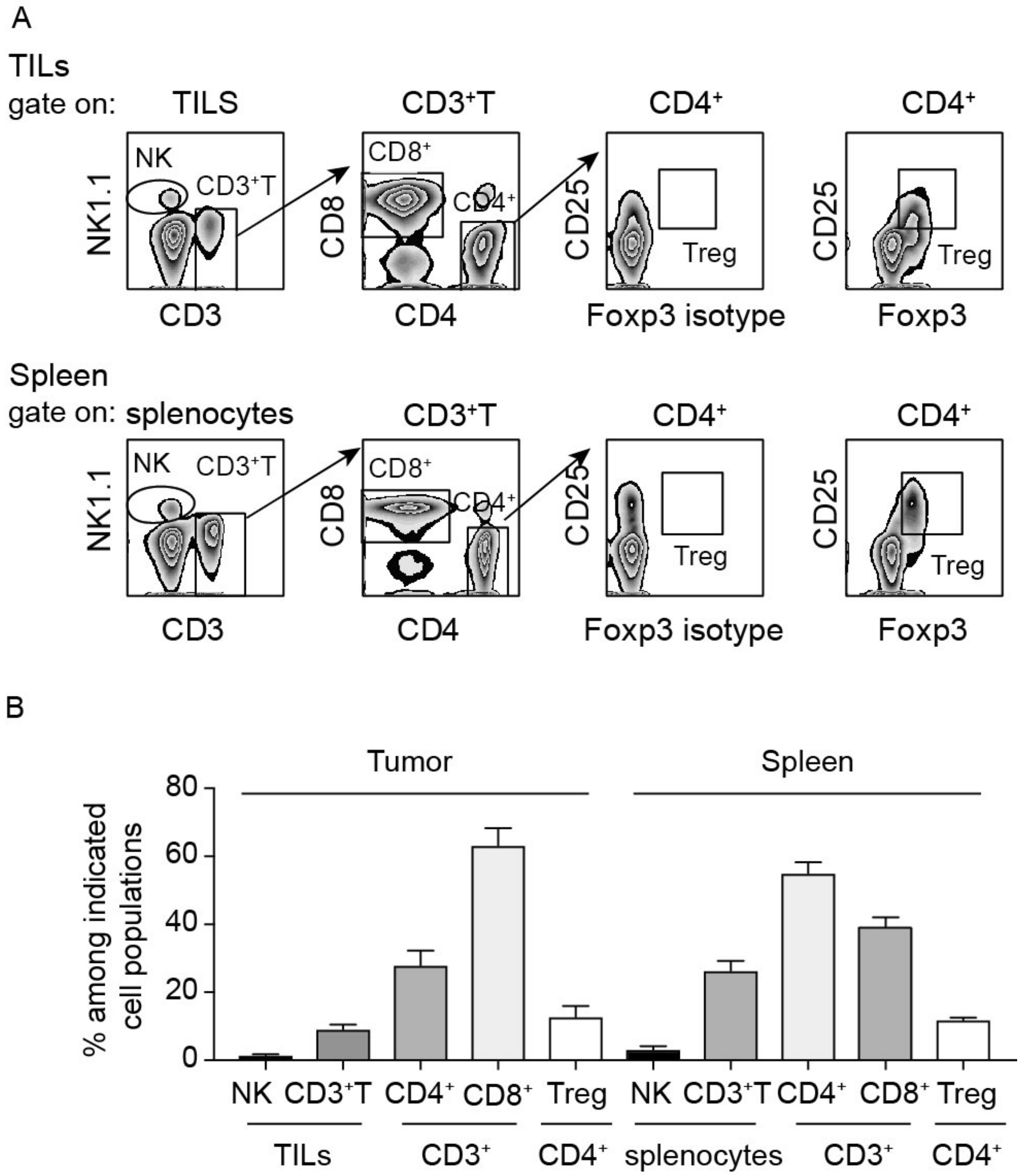

Figure S6 Lymphocyte populations in HCC tumor microenvironment. Tumor infiltrating leukocytes (TILs) and splenocytes were subjected to staining of surface markers (CD3, NK1.1, CD4, CD8α, CD25) and intracellular Foxp3, followed by flow cytometric analysis. (**A**) Gating for flow cytometric analysis. (**B**) Percentages of indicated cell populations among TILs, $CD3^+$ T cells, $CD4^+$ T cells, or splenocytes were shown. (n=7 mice).
